# *Tripterygium* glycosides sensitizes cisplatin chemotherapeutic potency by modulating gut microbiota in epithelial ovarian cancer

**DOI:** 10.3389/fcimb.2023.1236272

**Published:** 2023-09-25

**Authors:** Xinlu Zhan, Qi Zuo, Genhua Huang, Zhanghua Qi, Yufan Wang, Sihong Zhu, Yanying Zhong, Yifei Xiong, Tingtao Chen, Buzhen Tan

**Affiliations:** ^1^ Department of Obstetrics & Gynecology, The Second Affiliated Hospital of Nanchang University, Nanchang, China; ^2^ Department of Obstetrics & Gynecology, Ji’an Central People’s Hospital, Ji’an, China; ^3^ Institute of Translational Medicine, Nanchang University, Nanchang, China

**Keywords:** GTW, gut microbiota, chemosensitization, Lactobacillus acidophilus, intestinal barrier

## Abstract

Epithelial ovarian cancer (EOC) is a fatal gynecological malignancy with limited therapeutic options. Previous research has demonstrated that *Tripterygium* glycosides (GTW) can enhance effectiveness of cisplatin (DDP) chemotherapy against EOC. However, the underlying mechanism of GTW alleviating EOC still remains unclear. In this article, an ID8 cell-derived xenograft mouse model was established to evaluate the anti-tumor efficacy of GTW combined with DDP. Consistent with previous findings, the results suggested that GTW combined with DDP can exhibit a stronger tumor suppressive effect than DDP alone. Additionally, GTW was found can further exert gastrointestinal protection against DDP by reducing pathological damage on colon tissue. Secondly, to verify whether gut microbiota play an instrumental role in GTW’s anticancer effect, we treated mice models with antibiotic to eliminate gut microbiota. And our experimental results indicated that all drug groups showed a weaker tumor suppressive effect and more severe gastrointestinal damage post antibiotic supplement. At genus level, the relative abundance of *Lactobacillus* was dramatically diminished by the antibiotic treatment, while combined treatment of GTW and DDP can significantly restore the level. Moreover, we performed *Lactobacillus acidophilus* transplantation and healthy mice fecal microbiota transplantation experiments to further investigate the link between the anticancer effect of GTW and gut microbiota. Our results suggested that both cisplatin-sensitizing and intestinal barrier-protecting effects of GTW can be recovered to a different extent. In conclusion, our results indicated that GTW is a promising chemosensitization and intestinal barrier repair drug for EOC, and the potential mechanism may corelate with the restoration of the compromised intestinal microbial balance.

## Introduction

Epithelial ovarian cancer (EOC) is concerned as the most lethal gynecological malignancy worldwide, accounting for over 90% of total ovarian cancer (OC) (Kuroki and Guntupalli, 2020). Since the early-stage of EOC often lacks specific clinic symptoms, more than two-thirds of patients cannot be diagnosed until the cancer has progressed to an advanced stage, leading to a poor five-year survival rate of less than 30% (Penny, 2020). Current recommended standard EOC regimen is the combination of surgery followed by platinum-based chemotherapy, whereas those chemotherapeutic agents like cisplatin and paclitaxel can seriously threaten the health of EOC patients, causing peripheral neurotoxicity, nephrotoxicity and gastrointestinal mucositis *etc*. Therefore, seeking an innovative chemotherapeutic drugs or adjuvants that can improve platinum sensitivity has become an urgent requirement.

In recent years, Traditional Chinese Medicine (TCM), especially natural products, has become an important source of novel anticancer drugs, with advantages of weak toxicity and multi-targeted activity. *Tripterygium* glycosides (GTW), a prescription medicine, encompass a blend of glycosides extracted from the root of *Tripterygium wilfordii* Hook F. This botanical extract has been extensively utilized in treatments for nephritis, rheumatoid arthritis and systemic lupus erythematosus, and recent studies also indicated that GTW demonstrated a positive effects on liver cancer (Yang et al., 2019b), glioblastoma (Wang et al., 2020) and pancreatic cancer (Zhao et al., 2020). In our previous work, we revealed that GTW can inhibit invasiveness and metastasis of the cisplatin (DDP)-resistant EOC cell lines (A2780/DPP and SKOV3/DDP) and can also re-sensitize EOC to DDP via inhibition of epithelial-mesenchymal transition (EMT) and ILK/AKT/GSK3β/Slug pathway *in vivo* and *in vitro* (Feng et al., 2021; Yu et al., 2022). However, the specific mechanism underlying its antitumor efficacy in treating EOC still remains to be fully elaborated.

Gut microbiota, as a new research hotspot, play a crucial role in initiation and progression of diverse cardiovascular diseases, neurodegenerative diseases, metabolic diseases, and digestive diseases (Chen et al., 2021). With increasing application of high-throughput sequencing technology, a growing number of specific gut bacteria were found to be associated with cancers in multiple aspects. For instance, Federica et al. applied 16S ribosomal RNA (rRNA) high-throughput sequencing to investigate intestinal microbiome of 24 women with EOC and other 24 healthy individuals, and found Coriobacteriaceae were significantly enriched, whereas Lachnospiraceae were significantly decreased in EOC patients (D’Amico et al., 2021). Furthermore, based on another clinical trial, the abundance of Firmicutes and Bacteroidetes post-chemotherapy was increased in OC patients compared to prior treatment, while the abundance of Proteobacteria decreased (Tong et al., 2020). Additionally, novel therapeutic approaches such as probiotic supplementation and fecal microbiota transplantation (FMT) have now been proved can elevate the efficacy of chemotherapy (Ling et al., 2022). For instance, FMT attained from blank-treated mice can reduce chemoresistance and prolong the survival span of EOC-bearing mice, emphasizing the importance of an intact gut microbiome in acting as a tumor suppressor (Chambers et al., 2022). Given these findings, the vital role of intestinal microbiota in growth and advancement of cancer highlights its potential as a therapeutic target for treating EOC.

GTW, an orally administered drug, is bound to be closely linked to the alteration of intestinal microbiota. Our study aimed to evaluate whether GTW can enhance the effectiveness of chemotherapy for EOC through modulation of intestinal microbiota. To this end, an EOC xenograft model of ID8 cells was established. The potential anticancer effect of GTW in combination with DDP was estimated by analyzing tumor volumes, examining pathological changes in tumor tissues and measuring serum tumor marker levels. Alternations in intestinal microbiota were detected by 16S rRNA high-throughput sequencing. Our findings may provide valuable insights for the use of GTW as a promising adjuvant chemotherapy drug for future EOC treatment.

## Materials and methods

### Cell culture

ID8 cells, procured from American Type Culture Collection (ATCC), were cultured in Dulbecco’s Modified Eagle Medium (Gibco, 11875-085) supplemented with 10% heat-inactivated fetal bovine serum (VWR, 1500-500) and 1% penicillin-streptomycin (Gibco, 15140-122). Cells were incubated at 37°C, 5% CO_2_ with saturated humidity. Fresh medium was replaced every other day and, upon reaching 80-90% confluence, the cells were then washed, trypsinized, resuspended, and adjusted to produce a solution with density of 5 × 10^6^ cells per mL for later use.

### Lactobacillus acidophilus culture

The *Lactobacillus acidophilus* strain (NCU0082Chen, Laboratory of Translation Medical College, Nanchang University) was previously identified and isolated from feces of healthy women. The strains were stored in MRS media with 30% glycerol at −80°C. *L. acidophilus* was grown in MRS medium (37°C, 5% CO_2_, 48 h).

### Animal experiment

Female C57BL/6 mice (eight-week-old) were purchased from SJA Laboratory Animal Co., Ltd (Hunan, China), and were confined under 12 h light/12 h dark cycle and supplied with unlimited water and standard laboratory rodent food. Sterilized cages, water, and food were all autoclaved prior to use. Animal ethics was approved by the Laboratory Animal Ethics Committee of Nanchang Royo Biotechnology Co. Ltd (Ethics Number: RYE2021022501). All experimental animal procedure were conducted abiding National Institutes of Health Guide for the Care and Use of Laboratory Animals.

EOC xenograft model was constructed by injecting 5×10^6^ ID8 cells subcutaneously into the flank of mice. Tumor volumes were assessed at three-day intervals using vernier calipers. Randomization and administration were initiated upon the attainment of a tumor volume of 50 mm^3^.

**Figure 1:**
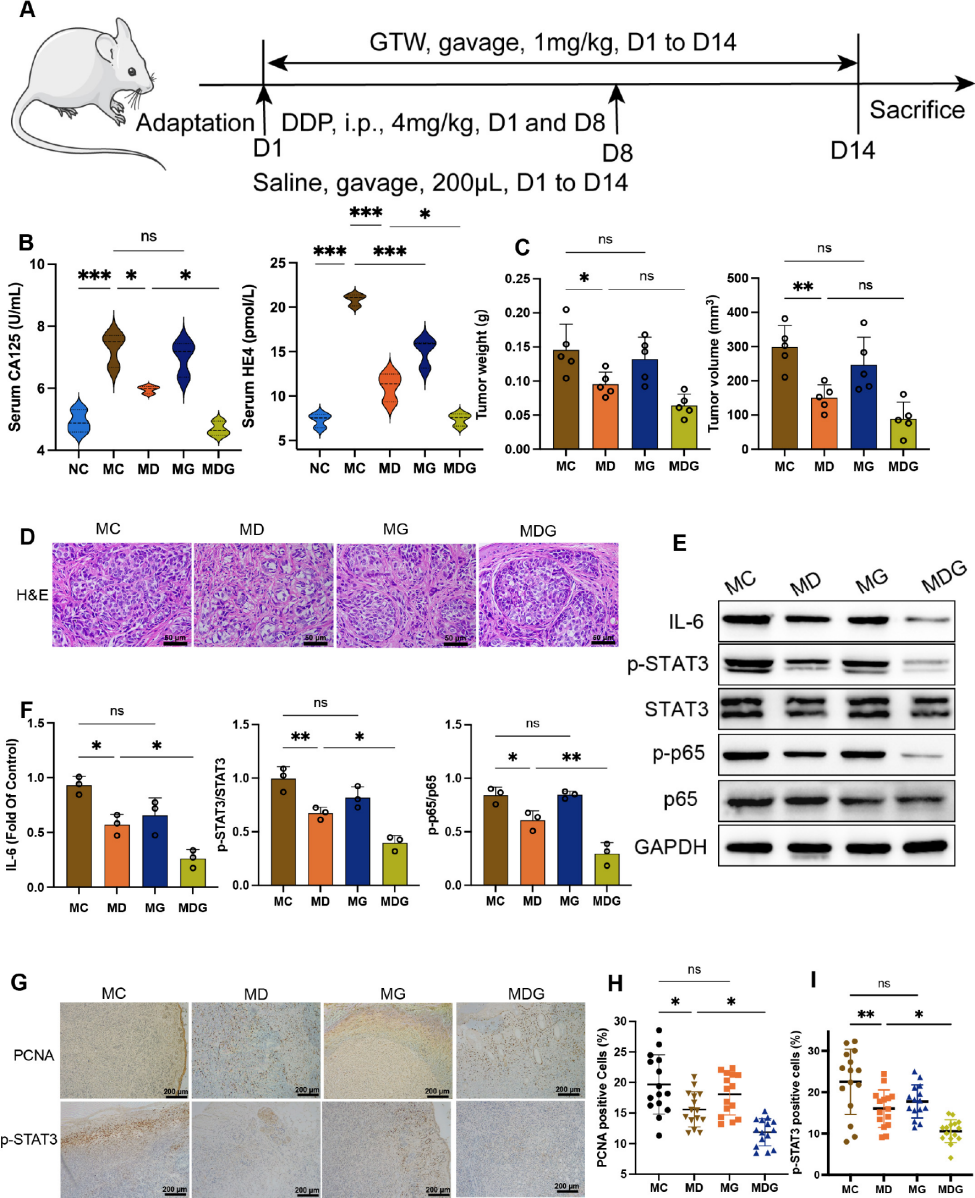
**(A)** Schematic of schedule of whole experiment. **(B)** CA125 and HE4 level in serum (n = 3). **(C)** Weights and volumes of tumours in different treatment groups. **(D)** Representative H&E staining image in tumour tissues (400x). Scale bsars, 50 µm. **(E)** Effect of different drugs on the protein expression in tumour tissues (n = 3). **(F)** Relative expression of IL-6, p-STAT3/STAT3 and p-p65/p65 in tumour tissues by ImageJ software (n = 3). **(G)** Representative immunohistochemical expression of PCNA and p-STAT3 in tumour tissues (100x). Scale bars, 200 µm. **(H, I)** Statistic analysis of PCNA and p-STAT3 positive cell expression in different drug groups. Five fields of three tumour sections from each group were randomly selected and examined. *p < 0.05, **p < 0.01, ***p < 0.001. NC stands for normal control group, MC stands for model control group, MD stands for model + DDP group, MG stands for model + GTW group, MDG stands for model + DDP + GTW group. ns, no significance.

In study of GTW treatment (**Figure 1A**), 50 mice were enrolled and equally randomized into five groups as followed: normal control group (NC group), healthy mice were gavage 200 μL of 0.9% saline once per day from day 1 to day 14, n = 10; model control group (MC group), xenograft tumor model mice were gavage with 200 μL of 0.9% saline once per day from day 1 to day 14, n = 10; model + DDP group (MD group), model mice were gavage with 200 μL of 0.9% saline once per day from day 1 to day 14, and were applied with intraperitoneal injection of DPP (4 mg/kg) at day 1 and day 8, once per day, n = 10; model + GTW group (MG group), model mice received GTW via oral administration at dose of 1 mg/kg once per day from day 1 to day 14, n = 10; and model + DDP + GTW group (MDG group), model mice were gavage with GTW (1 mg/kg) once per day from day 1 to day 14, and also intraperitoneally injected with DDP (4 mg/kg) at day 1 and day 8, once per day, n = 10. GTW (Gleep Company, China) was diluted from stock (dissolved in 0.1% DMSO) in 0.9% saline; the administration dose of GTW was 1 mg/kg. DDP was purchased from Hanson Pharma (China); the intraperitoneal injection dose of DDP was 4 mg/kg.

**Figure 2:**
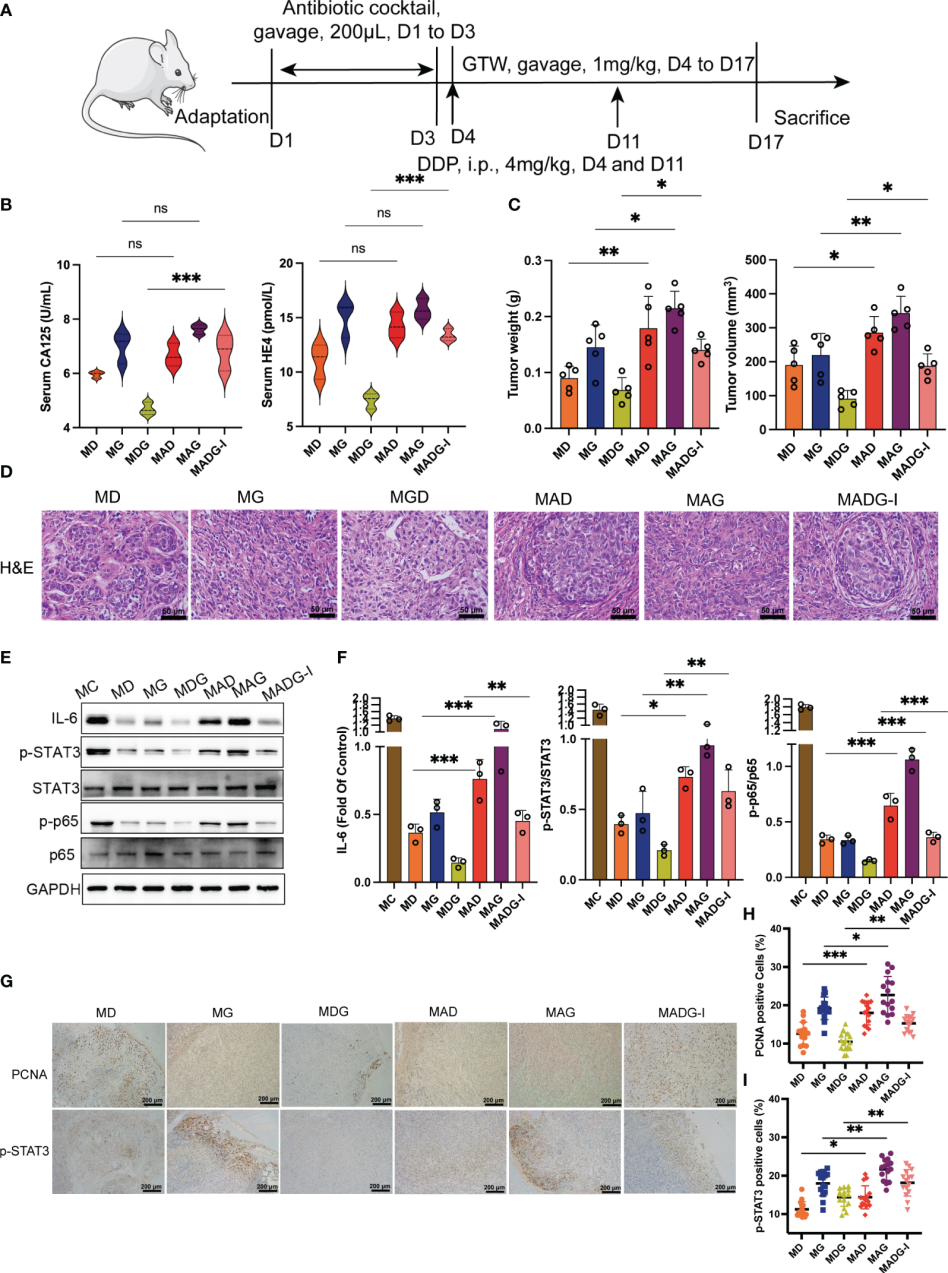
**(A)** Schematic of schedule of whole experiment. **(B)** CA125 and HE4 level in serum (n = 3). **(C)** Weights and volumes of tumours in different treatment groups. **(D)** Representative H&E staining image in tumour tissues (400x). Scale bars, 50 µm. **(E)** Effect of different drugs on the protein expression in tumour tissue (n = 3). **(F)** Relative expression of IL-6, p-STAT3/STAT3 and p-p65/p65 in tumour tissues by ImageJ software (n = 3). **(G)** Representative immunohistochemical expression of PCNA and p-STAT3 in tumour tissues (100x). Scale bars, 200 µm. **(H, I)** Statistic analysis of PCNA and p-STAT3 positive cell expression in different drug groups. Five fields of three tumour sections from each group were randomly selected and examined. *p < 0.05, **p < 0.01, ***p < 0.001. MC stands for model control group, MD stands for model + DDP group, MG stands for model + GTW group, MDG stands for model + DDP + GTW group, MAD stands for model + antibiotic +DDP group, MAG stands for model +antibiotic + GTW group, MADG-I stands for model + antibiotic +DDP + GTW group. ns, no significance.

In study of gut microbiota and GTW (**Figure 2A**), 40 model mice were administered with pretreatment of 200 μL of 0.9% saline once per day from day 1 to day 3. Mice were then randomly divided into following four groups: MC group received an oral gavage of 200 μL of 0.9% saline once per day from day 4 to day 17, n = 10; MD group received an oral gavage of 200 μL of 0.9% saline once per day from day 4 to day 17 and an intraperitoneal injection of DPP (4 mg/kg) at day 4 and day 11, once per day, n = 10; MG group received an oral gavage of 200 μL GTW at dosage of 1 mg/kg once per day from day 4 to day 17, once per day, n = 10; and MDG group received treatment with both DDP and GTW, n = 10. And additional 30 model mice were pretreated with daily oral gavage of 200 μL of antibiotic cocktail once a day from day 1 to day 3, with 6 h fast after each daily antibiotic administration. After above pretreatment, fecal samples were collected from model + antibiotic group (MA group) for further experimentation. These 30 model mice pretreated with antibiotic cocktail were then randomly assigned into three groups: model + antibiotic + DDP group (MAD group) received dosing with DDP identical to that of the MD group, n = 10; model + antibiotic + GTW group (MAG group) received dosing with GTW identical to that of the MG group, n = 10; and model + antibiotic + GTW + DDP group (MADG-I group) received dosing with both DDP and GTW identical to that of the MDG group, n = 10. Antibiotic cocktail comprised 0.5 g/L vancomycin, 1 g/L ampicillin, 1 g/L metronidazole and 1 g/L neomycin, dissolved in normal saline.

**Figure 3:**
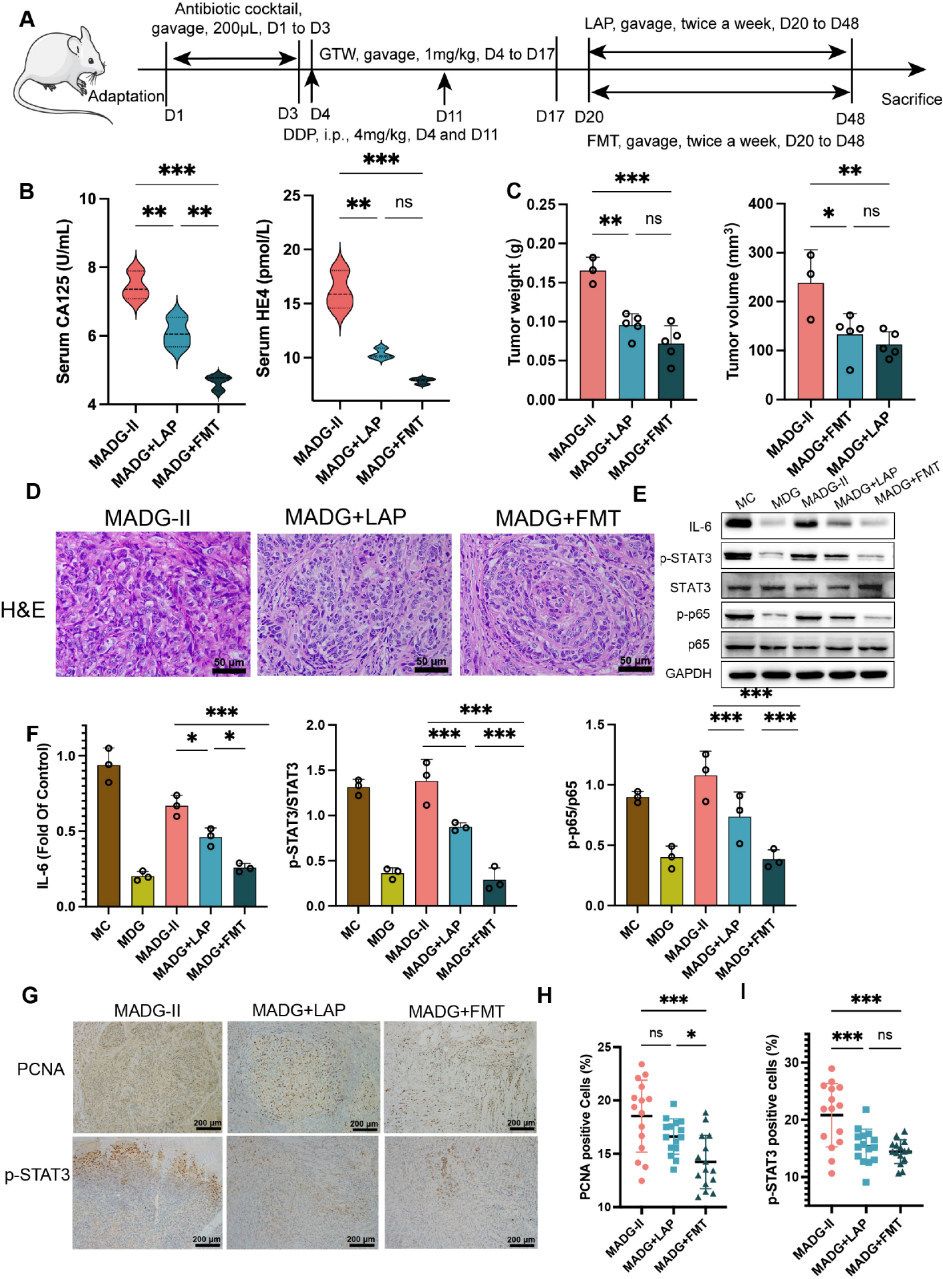
**(A)** Schematic of schedule of whole experiment. **(B)** CA125 and HE4 level in serum (n = 3). **(C)** Weights and volumes of tumours in different treatment groups. **(D)** Representative H&E staining image in tumour tissues (400x). Scale bars, 50 µm. **(E)** Effect of different drugs on the protein expression in tumour tissues (n = 3). **(F)** Relative expression of IL-6, p-STAT3/STAT3 and p-p65/p65 in tumour tissues by ImageJ software (n = 3). **(G)** Representative immunohistochemical expression of PCNA and p-STAT3 in tumour tissues (100x). Scale bars, 200 µm. **(H, I)** Statistic analysis of PCNA and p-STAT3 positive cell expression in different drug groups. Five fields of three tumour sections from each group were randomly selected and examined. *p < 0.05, **p < 0.01, ***p < 0.001. MADG-II stands for model + antibiotic +DDP + GTW group, MADG + LAP stands for model + antibiotic + GTW + DDP + *L. acidophilus* group, MADG + FMT stands for model + antibiotic + GTW + DDP + fecal bacteria transplantation group. ns, no significance.

In study of *L. acidophilus* supplementation and fecal microbiome transplantation (**Figure 3A**), 30 model mice were divided into three groups with randomization: In MADG-II group, model mice were given 200 μL of an antibiotic cocktail once per day from day 1 to day 3 orally, followed by dosing with GTW and DDP identical to that of previous MDG group from day 4 to day 17. Subsequently, the mice were then given 200 μL of 0.9% saline twice a week from day 20 to day 48. In the model + antibiotic + GTW + DDP + *L. acidophilus* group (MADG + LAP group), model mice received the same antibiotic cocktail and dosing of GTW and DDP as the MADG-II group from day 1 to day 17, followed by gavage 200 μL of pure *L. acidophilus* at a dose of 1×10^8^ colony forming units (CFU), suspended in 0.9% saline, twice a week from day 20 to day 48. In the model + antibiotic + GTW + DDP + fecal bacteria transplantation group (MADG + FMT group), model mice received the same antibiotic cocktail and dosing of GTW and DDP as the MADG-II group from day 1 to day 17, followed by gavage of 200 μL of fecal supernatant from normal mice 2 times a week from day 20 to day 48. Unfortunately, seven mice from the MADG-II group were found dead before the end of the experiment. The fecal suspension was prepared as followed: fresh fecal pellets were collected from NC group, and were immediately weighed and suspended in 0.9% saline, then resuspended with a vortex. After resuspension, microcentrifuge tubes containing feces in 0.9% saline were centrifuged for 5 min at 3,000 rpm to remove impurities.

At the end of the experiment, mice were anesthetized with isoflurane, and stool, tumor, serum and colon samples were collected and frozen at −80°C immediately until subsequent analysis.

### 16S rRNA gene V4 region sequencing analysis

Fresh fecal samples were obtained from MC group (n = 7), MA group (n = 7), MADG-I group (n = 7), MADG-II group (n = 3), MADG + FMT group (n = 7) and MADG + LAP group (n = 7). A DNA extraction kit (Tiangen, China) was used to extract genomic DNA from the samples.

Paired-end sequencing was performed on the Illumina HiSeq 6000 platform followed by analysis using the free online platform (https://www.genescloud.cn/home). The ASV/OTU signature sequences were then obtained using the DADA2 method. ASV/OTU data were used to analyze the species composition, alpha-diversity, beta-diversity and species difference. The raw data from sequencing were uploaded in the NCBI sequence read archive (SRA) under the BioProject ID PRJNA778571.

### Serum analysis

Blood samples were placed at room temperature for a minimum of 2 h, followed by centrifuged at 3,000 rpm for 15 min at 4°C. Subsequently, serum was then stored at −80°C. Next, the enzyme-linked immunosorbent assay (ELISA) kits for mice (Mlbio, China) were employed to measure the serum levels of HE4 and CA-125, both of which are recognized biomarkers of OC(Sun et al., 2022).

### Histological analysis

Tumor and colon tissues were embedded and sliced into 6 μm-thick sections. Haematoxylin-eosin (H&E) staining was performed using an H&E staining kit (Solarbio, China), according to the manufacturer’s instructions.

For immunohistochemistry staining, tumor sections were incubated with primary antibodies such as PCNA (1:4000, CST, #13110) and phospho-STAT3 (1:400, CST, # 9145S) for 2 h at 37°C. After washing with PBS three times, the sections were incubated with secondary antibodies at room temperature for 1 h. Visualization of the staining was achieved using diaminobenzidine (DAB). Five fields of high magnification were selected for each slice randomly to detect the average absorbance (three slices from each group).

### Western blotting analysis

Tumor and colon tissue were placed into a centrifuge tube, and RIPA lysis buffer (Thermo, #89901) and protease and phosphatase inhibitor cocktail (Thermo, #78442) were then added. All operations were performed on ice. Total protein concentrations were uniformized after quantification using the BCA kit (Thermo, #23227). Protein was then separated on 6%~10% separator gel and 5% concentrate glue and transferred onto a 0.22 µm polyvinylidene fluoride membrane. Membranes were blocked in 5% skimmed milk (CST, #9999) in Tris-buffered saline with 0.1% Tween 20 (TBST) at room temperature for 90 min. The primary antibody was added into Primary Antibody Dilution Buffer (Applygen, China) to adjust an appropriate concentration. After undergoing three 10 min washes with TBST, membranes were then subjected to overnight incubation at 4°C with the primary antibody. Then, membranes were washed with TBST for 10 min, three times, and incubated with 5% bovine serum albumin (BSA)-TBST diluted secondary antibody for 90 min at room temperature. The protein bands were detected using an enhanced chemiluminescence (ECL) kit (Thermo, #32209).

The following primary antibodies were used: rabbit anti-IL-6 (1:1000, CST, #12912), rabbit anti-NF-κB p65 (1:1000, CST, #8242), rabbit anti-phospho-NF-κB p65 (Ser536) (1:1000, CST, #3033), mouse anti-STAT3 (1:1000, CST, #9139), rabbit anti-phospho-STAT3 (1:1000, CST, #9145), rabbit anti-ZO-1 (1:1000, CST, #8193), rabbit anti-occludin (1:1000, CST, #91131) and mouse anti-GAPDH (1:10000, Proteintech, 60004-1-Ig).

### Statistical analysis

GraphPad Prism 9.0 software was used for statistical analysis. The mean ± standard deviation (SD) was used to present the measurement data. Differences between two groups were compared using unpaired *t*-tests. Comparisons among more than two groups were performed using one-way ANOVA followed by Tukey’s *post hoc* test. A *p* value < 0.05 was considered statistically significant in this experiment. To ensure reliability, all experiments were repeated a minimum of three times.

## Results

### GTW synergistically enhances the antitumor effect of DDP and reduces gastrointestinal cytotoxicity

To explore the possible chemosensitization effect of GTW, a xenograft mouse model of ID8 cell was established, the whole experimental schedule is shown in **Figure 1A**. In MC group, the concentration of CA125 (7.293 ± 0.5445 vs. 4.927 ± 0.3646 U/mL, *p <* 0.001) and HE4 (20.860 ± 0.6455 vs. 7.305 ± 0.7881 pmol/L, *p <* 0.001) in serum were significantly elevated compared to NC group (**Figure 1B**). GTW treatment alone led to a reduction in the serum levels of HE4 (*p <* 0.001), while did not exert a significant impact on the serum levels of CA125 (*p >* 0.05) in comparison to MC group. Compared to MC group, the serum levels of HE4 (*p <* 0.001) and CA125 (*p <* 0.05) in MD group were significantly decreased. Moreover, the combination therapy group, MDG group, demonstrated a significant reduction in HE4 (*p <* 0.05) and CA125 (*p <* 0.05) levels compared to MD group.

DDP alone, as well as the combined treatment with DDP and GTW, significantly inhibited the tumor volume (150.5 ± 38.1 mm^3^ in the MD group vs. 89.0 ± 48.6 mm^3^ in the MDG group, 298.7 ± 63.2 mm^3^ in the MC group) and tumor weight (0.095 ± 0.018 g in the MD group vs. 0.064 ± 0.017 g in the MDG group, 0.146 ± 0.038 g in the MC group) compared with those parameters in MC group (**Figure 1C**). However, administration of GTW alone did not reduce tumor volume (*p >* 0.05) or tumor weight (*p >* 0.05). H&E staining revealed that tumor cells in MC group were denser and more compact, whereas the tumor construct was loosely distributed in MD and MG groups, with more mesenchymal infiltration (**Figure 1D**). This variation is more evident upon comparing MDG group with MD group.

To further investigate the photosensitizing mechanism of GTW, we used Western blotting to examine the key proteins involved in NF-κB/IL-6/STAT3 signaling pathway in tumor tissues, including IL-6, NF-κB (p65), phospho-NF-κB (p-p65), STAT3, phospho-STAT3 (p-STAT3) (**Figure 1E**). Compared to MC group, IL-6 protein levels (0.5713 ± 0.0941 vs. 0.9316 ± 0.0808, *p <* 0.05), p-STAT3/STAT3 protein ratio (0.6727 ± 0.0567 vs. 0.9943 ± 0.1119, *p <* 0.01) and p-p65/p65 ratio (0.6074 ± 0.0881 vs. 0.8420 ± 0.0753, *p <* 0.05) in MD group decreased significantly (**Figure 1F**). In contrast, there was no significant difference between MG and MC groups in terms of IL-6 protein levels, p-STAT3/STAT3 protein ratio and p-p65/p65 ratio. Simultaneously, MDG group showed a marked reduction in overall IL-6 levels (*p <* 0.05), as well as p-STAT3/STAT3 protein ratio (*p <* 0.05) and p-p65/p65 ratio (*p <* 0.01) compared to MD group. STAT3 and p65 protein levels were similar among all four groups.

Immunohistochemistry was performed to evaluate the expression levels of the proliferative marker PCNA and p-STAT3 (**Figure 1G**). We found the expression level of PCNA and p-STAT3 was decrease greatly in MDG group (*p <* 0.05 and *p <* 0.05) compared with MD group (**Figures 1H, I**), indicating the inhibition of tumor proliferation and NF-κB/IL-6/STAT3 signaling downstream protein.

**Figure 4:**
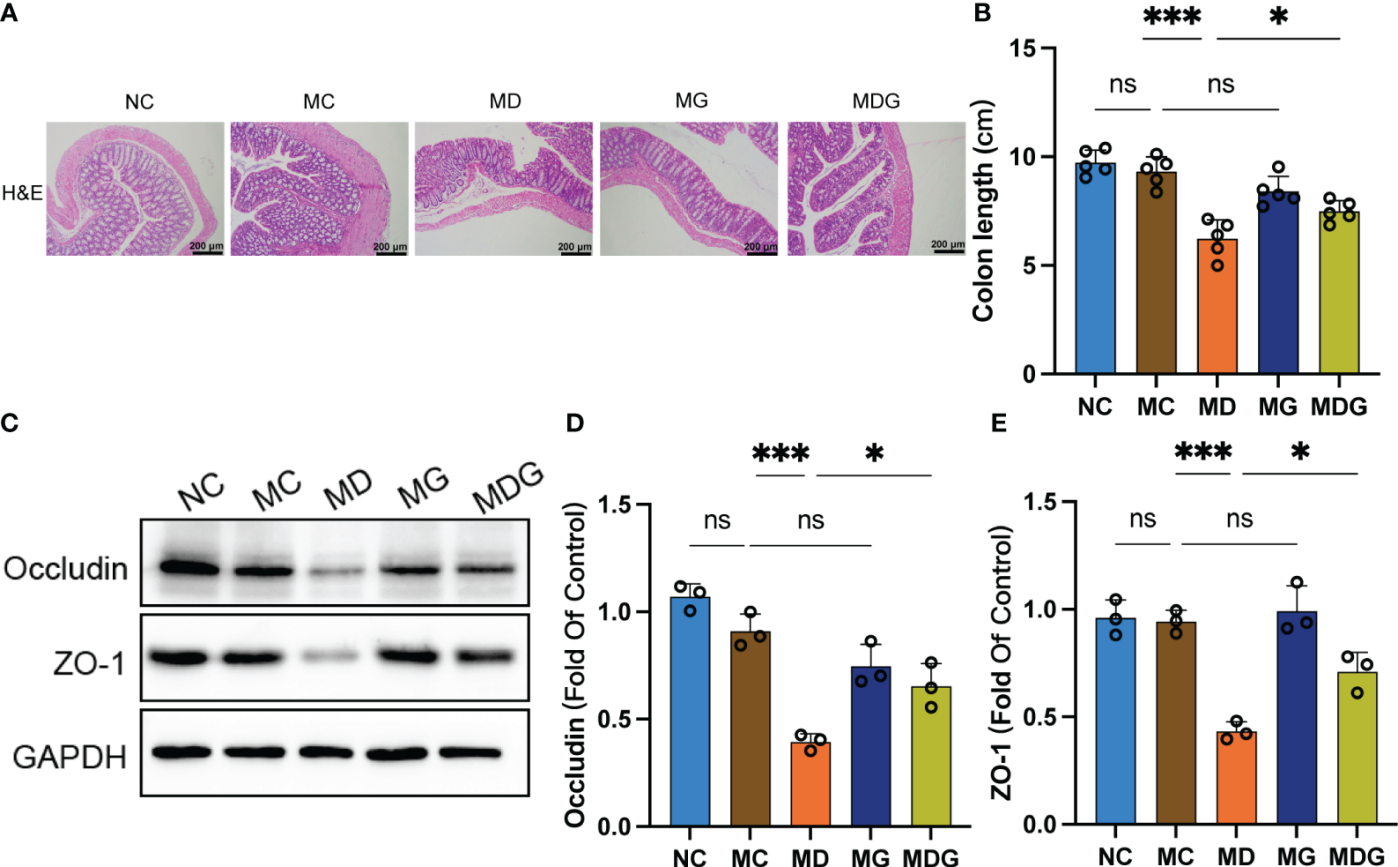
**(A)** Representative H&E staining image in colon tissues (100x). Scale bars, 200 µm. **(B)** Colon length. **(C)** Effect of different drugs on the protein expression in colon tissues (n = 3). **(D, E)** Relative expression of occludin and ZO-1 in colon tissues (n = 3). *p < 0.05, ***p < 0.001. NC stands for normal control group, MC stands for model control group, MD stands for model + DDP group, MG stands for model + GTW group, MDG stands for model + DDP + GTW group. ns, no significance.

A common and irritating adverse effect in DDP chemotherapy is epithelial damage in the gastrointestinal tract (Shahid et al., 2018). To investigate whether GTW can alleviate the gastrointestinal cytotoxicity induced by DDP chemotherapy, we investigated the incidence of pathological changes, colon lengths, and tight junction proteins in the colonic tissue. Colon tissue from NC and MC groups did not exhibit any specific pathologic changes. However, when compared with MC group, it was found that MD group seemed to have compromised epithelial surface in colon, thinner mucosa, reduced glands, decreased goblet cell numbers, and inflammatory cells’ infiltration into the lamina propria. This histological damage was significantly ameliorated in MDG group (**Figure 4A**). Additionally, the colon lengths of MD group were significantly reduced (*p <* 0.001) compared to MC group. While single-agent GTW treatment did not significantly alter colon lengths, whereas the combination of GTW and DDP was viable to revert the colon lengths (*p <* 0.05) compared to DDP treatment group (**Figure 4B**).

Subsequently, we used Western blotting to quantify the expression level of zonula occludens-1 (ZO-1) and occludin in colon tissues, which are essential proteins to maintain tight junction stability and barrier function (**Figure 4C**). As shown in **Figures 4D, E**, no significant differences were observed on occludin and ZO-1 protein expression between NC group and MC group. However, protein levels of occludin (0.3944 ± 0.0378 vs. 0.9095 ± 0.0800, *p <* 0.001) and ZO-1 (0.4335 ± 0.0431 vs. 0.9426 ± 0.0532, *p <* 0.001) were markedly downregulated in MD group than MC group, while no significant change was observed in MG group. Notably, the combination of DDP and GTW restored the abundance of occludin (0.6546 ± 0.1042 vs. 0.3944 ± 0.0378, *p <* 0.05) and ZO-1 (0.7111 ± 0.0877 vs. 0.4335 ± 0.0431, *p <* 0.05) significantly when compared with DDP single-agent group.

These results suggest that the administration of GTW may enhance the anticancer effect of DDP while alleviating the damage to gut caused by DDP.

### Cisplatin sensitization and intestinal protective benefits of GTW were correlated with gut microbiota

Previous research have revealed a significant correlation between the efficacy of antitumor therapeutic response and the gut microbiome (Liu and Shah, 2022). To investigate potential correlations between chemotherapy drug effectiveness and gut microbiota, we depleted most of the microbiota by pretreating mice with antibiotic cocktail (**Figure 2A**).

As shown in **Figure 2B**, only the group treated with DDP, GTW and antibiotics (MADG-I group) exhibited a significant increase in serum CA125 (*p <* 0.001) and HE4 (*p <* 0.001) levels than the groups treated with DDP and GTW only (MDG group). Groups treated with chemotherapy drug combined with antibiotics (MAD group and MAG group) did not exhibit any significant changes in serum CA125 and HE4 compared to those treated in the single chemotherapy drug treatment groups (MD and MG). Tumor weight and volume is shown in **Figure 2C**, revealing that antibiotic pretreatment improved tumor growth, with both increased tumor volume and weight compared to other three chemotherapy drug treatment groups. H&E staining for tumor tissue pathology further revealed that antibiotic pretreatment can promote the proliferation of tumor cells (**Figure 2D**). Additionally, IL-6 protein levels, p-STAT3/STAT3 protein ratio and p-p65/p65 ratio were substantially increased in the groups using chemotherapy drugs combined with antibiotics compared to those groups receiving only chemotherapy drugs. STAT3 and p65 protein levels did not exhibit significant changes between each group (**Figures 2E, F**). Moreover, **Figures 2G–I** showed that the expression of PCNA and p-STAT3 were vastly upregulated in the groups treated with chemotherapy drug combined with antibiotics compared with those treated with chemotherapy drugs only.

**Figure 5:**
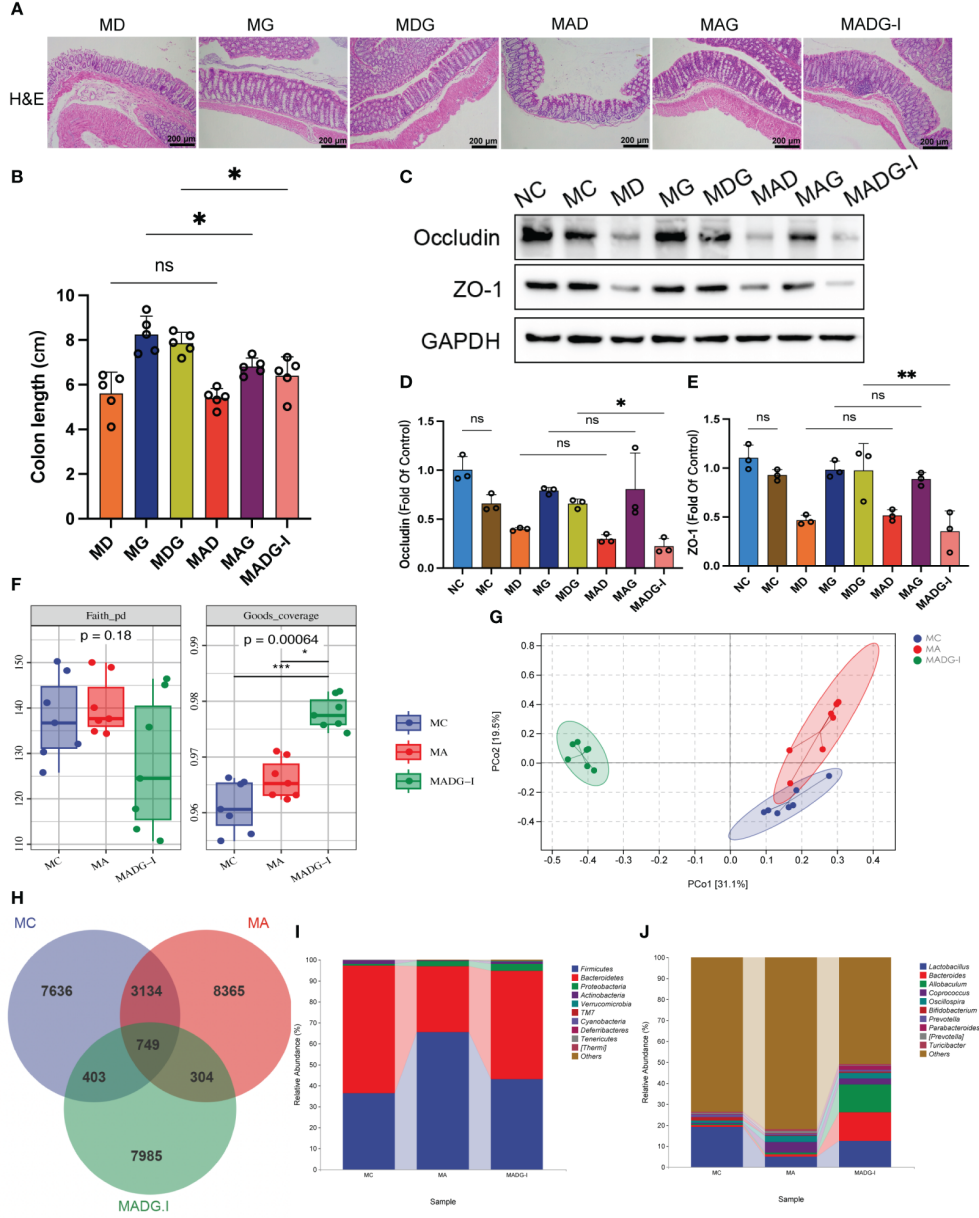
**(A)** Representative H&E staining image in colon tissues (100x). Scale bars, 200 µm. **(B)** Colon length. **(C)** Effect of different drugs on the protein expression in colon tissues (n = 3). **(D, E)** Relative expression of occludin and ZO-1 in colon tissues (n = 3). **(F)** Faith_pd and Goods_coverage indexes of the alpha diversity. **(G)** PCoA of the β-diversity index. **(H)** Venn map representing OTUs. **(I)** Relative abundance of bacteria at phylum level. **(J)** Relative abundance of bacteria at genus level. *p < 0.05, **p < 0.01, ***p < 0.001. NC stands for normal control group, MC stands for model control group, MA stands for model + antibiotic group, MD stands for model + DDP group, MG stands for model + GTW group, MDG stands for model + DDP + GTW group, MAD stands for model + antibiotic + DDP group, MAG stands for model + antibiotic + GTW group, MADG-I stands for model + antibiotic + DDP + GTW group. ns, no significance.

Compared with the single drug groups, the colonic histological damage was significantly aggravated by antibiotic treatment (**Figure 5A**), and the colon lengths were significantly reduced in MAG and MADG-I groups when compared with MG and MDG groups (**Figure 5B**). Furthermore, as demonstrated in **Figures 5C–E**, protein expression of occludin and ZO-1 were considerably downregulated in MADG-I group compared with MDG group.

We then used 16S rRNA V4 gene sequencing technology to evaluate the gut microbiota composition in mice from MC, MA and MADG-I groups (n = 7). Faith_pd index and Goods_coverage index were used to estimate the α-diversity of microbial communities. No significant differences were observed in Faith_pd index among the three groups. However, the Goods_coverage index in the MADG-I group obviously increased compared with MC and MA groups (**Figure 5F**). Principal coordinate analysis (PCoA) was used to compare the beta-diversity profiles across three groups (**Figure 5G**). Samples in MC and MA groups exhibited a certain degree of proximity to each other, while remained distinctly different from MADG-I group, indicating a variation in the microbial diversity between MADG-I group and either MC or MA group. Furthermore, we employed Venn diagram method to analyze these three groups, identifying a total of 749 common operational taxonomic units (OTUs). The unique OTU numbers in the MC, MA and MADG-I groups were 7,636, 8,365 and 7,985, respectively (**Figure 5H**). At phylum level, the most dominant two phyla were Firmicutes and Bacteroidetes. The Firmicutes/Bacteroidetes ratio increased significantly in MA group compared to MC group, but interestingly, this ratio restored into MC group level in MADG-I group (**Figure 5I**). At genus level, the abundance of *Lactobacillus*, *Bacteroides*, *Bifidobacterium*, *Prevotella* and *Parabacteroides* were decreased, whereas the abundance of *Allobaculum*, *Coprococcus*, *Oscillospira*, *[Prevotella]* and *Turicibacter* were increased in MA group compared with MC group. The abundance of *Coprococcus*, *Oscillospira*, *[Prevotella]* and *Turicibacter* were also decreased in MADG-I group compared to MA group, whereas the abundance of *Lactobacillus, Bacteroides*, *Allobaculum*, *Bifidobacterium*, *Prevotella* and *Parabacteroides* were increased (**Figure 5J**).

Overall, these data suggest that antibiotic pretreatment can significantly reduce the genus abundance of *Lactobacillus*, while the combination of DDP and GTW can restore its level. Of note, antibiotic pretreatment can disrupt the biological barrier of the intestine and limit the efficiency of chemotherapy. We next focused on the MDG group, which demonstrated the strongest anti-tumor effect.

### *L. acidophilus* supplementation and fecal microbiota transplantation can reduce intestinal damage and increase the efficacy of chemotherapy drugs

*L. acidophilus* is a classic representative strain in *Lactobacillus* genus and is also an extensively prescribed probiotic (Mital and Garg, 1995). Our clinical trials provided firm evidence that applying a probiotic combination, with *L. acidophilus* as the main ingredient, can effectively alleviate chemotherapy-induced gastrointestinal complications in colorectal cancer sufferers (Huang et al., 2023). Furthermore, it has been found that *L. acidophilus* supplementation can enhance intestinal epithelial function in the context of irradiation-induced intestinal damage (Sittipo et al., 2020). Given the significant correlation between chemotherapy efficacy and gut microbiota, we performed a *L. acidophilus-*transplanted (LAP) and fecal microbiota transplanted (FMT) mice model to further explore the role of gut microbiome in the chemotherapeutic response in GTW-based adjuvant treatment (**Figure 3A**). Unfortunately, at the end of the experiment (day 48), a total of seven mice in the MADG-II group had died.

As demonstrated in **Figure 3B**, a comparison of CA125 and HE4 serum levels between three groups suggested that both FMT (MADG + FMT group) and *L. acidophilus* transplantation (MADG + LAP group) were able to decrease the serum level of tumor markers, with FMT showing a greater effect on reducing CA125 levels than *L. acidophilus* transplantation. Further, MADG + FMT group and MADG + LAP group were able to inhibit tumor growth compared to MADG-II group, but no significant difference was observed between these two groups (**Figure 3C**). H&E staining of tumor tissue revealed that both *L. acidophilus* supplementation and FMT can inhibit the proliferation of tumor cells (**Figure 3D**). Compared to MADG-II group, MADG + LAP group and MADG + FMT group exhibited a significant reduction in the IL-6 protein levels, as well as a decrease in p-STAT3/STAT3 protein ratio and p-p65/p65 ratio, with the reduction in MADG + FMT group being more pronounced comparing to MADG + LAP group (**Figures 3E, F**). Additionally, the expression level of PCNA and p-STAT3 also showed a declining trend in MADG-II, MADG + LAP, and MADG + FMT groups (**Figures 3G–I**).

**Figure 6:**
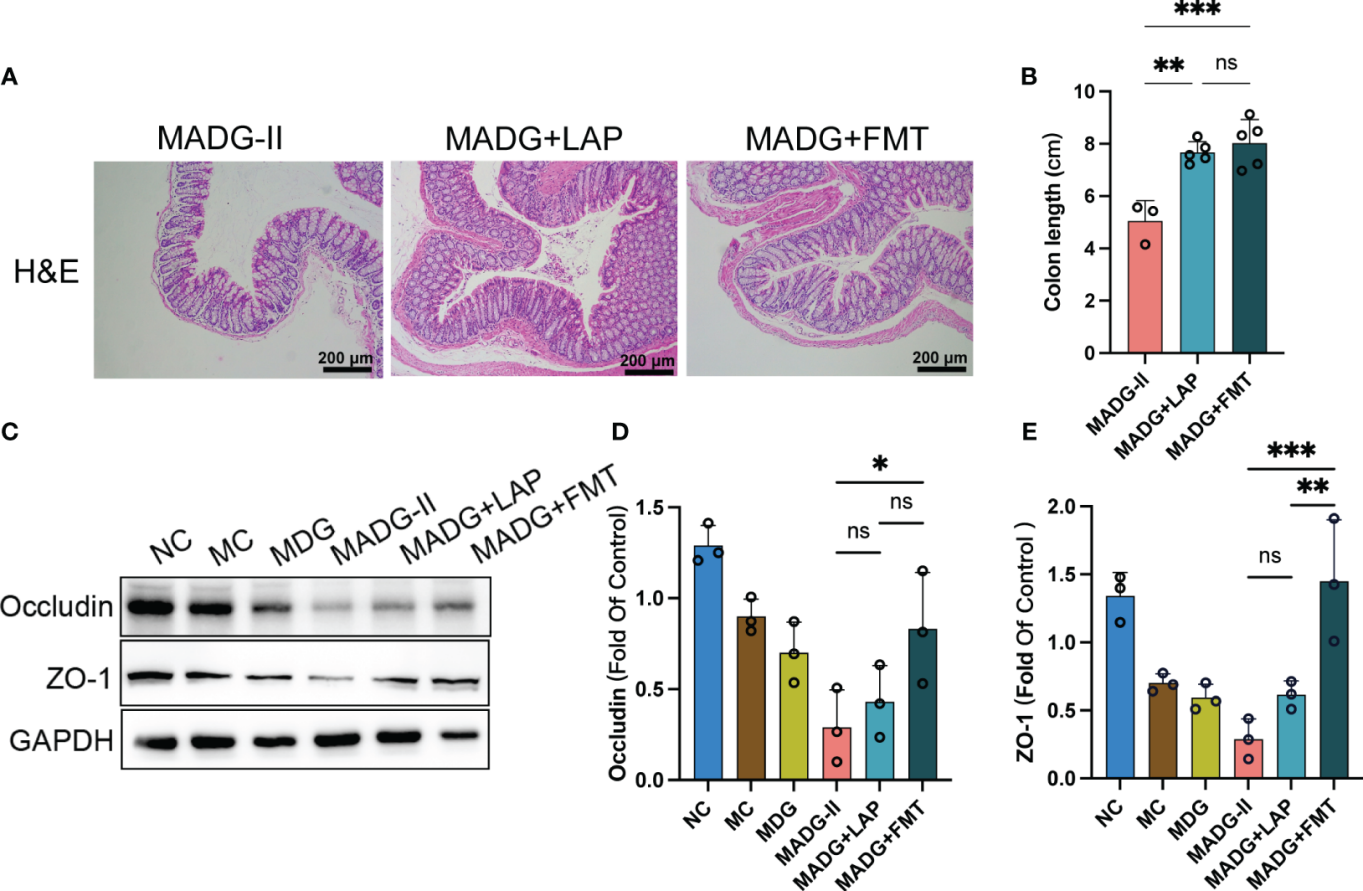
**(A)** Representative H&E staining image in colon tissues (100x). Scale bars, 200 µm. **(B)** Colon length. **(C)** Effect of different drugs on the protein expression in colon tissues (n = 3). **(D, E)** Relative expression of occludin and ZO-1 in colon tissues (n = 3). *p < 0.05, **p < 0.01, ***p < 0.001. NC stands for normal control group, MC stands for model control group, MDG stands for model + DDP + GTW group, MADG-II stands for model + antibiotic + DDP + GTW group, MADG + LAP stands for model + antibiotic + GTW + DDP + *L. acidophilus* group, MADG + FMT stands for model + antibiotic + GTW + DDP + fecal bacteria transplantation group. ns, no significance.

Meanwhile, FMT treatment showed a significant alleviation of colonic histological damage when compared to MADG-II group (**Figure 6A**), and colon lengths were significantly increased in MADG + LAP and MADG + FMT groups compared to MADG-II group (**Figure 6B**). Furthermore, the protein expression of occludin and ZO-1was only markedly restored in the MADG + FMT group compared with the MADG-II group (**Figures 6C–E**).

Collectively, these data suggested that both *L. acidophilus* transplantation and FMT can contribute to maintain the biological barrier of intestine and improve the efficiency of chemotherapy, which seems to be mainly associated with *L. acidophilus*.

### Effects of *L. acidophilus* supplementation and FMT on intestinal microbiota

To determine whether gut microbiota changes from *L. acidophilus* supplementation or FMT combined with DDP and GTW, we evaluated fecal microbial samples in MADG-II (n = 3), MADG + LAP (n = 7) and MADG + FMT groups (n = 7) by 16S rRNA analysis.

**Figure 7:**
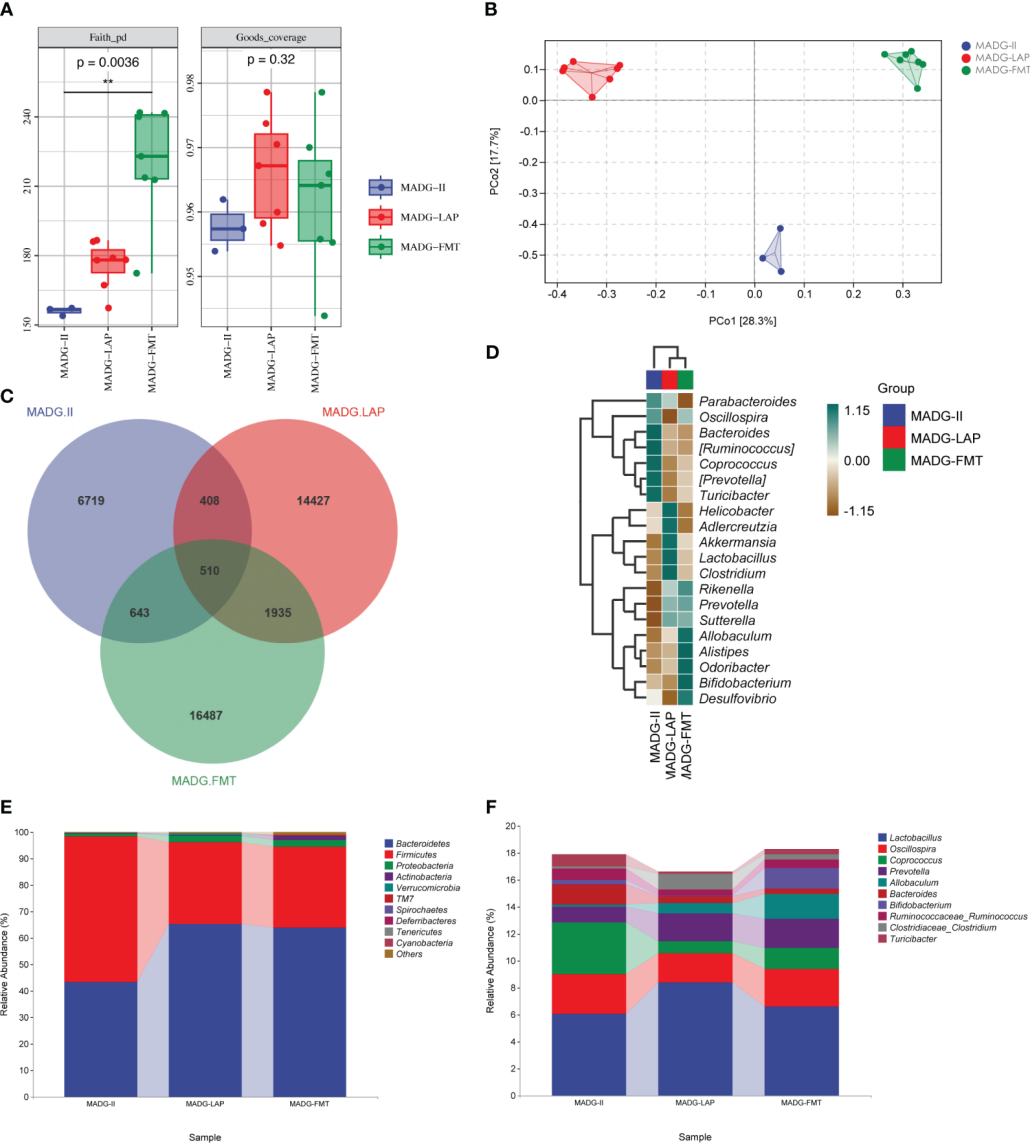
**(A)** Faith_pd and Goods_coverage indexes of the alpha diversity. **(B)** PCoA of the β-diversity index. **(C)** Venn map representing OTUs. **(D)** Heat map of the relative abundance of the top 20 genera. **(E)** Relative abundance of bacteria at the phylum level. **(F)** Relative abundance of bacteria at the genus level. Data are presented as mean ± SD. One-way repeated-measures ANOVA with Tukey’s test used for multiple comparisons. MADG-II stands for model + antibiotic + DDP + GTW group, MADG + LAP stands for model + antibiotic + GTW + DDP + *L. acidophilus* group, MADG + FMT stands for model + antibiotic + GTW + DDP + fecal bacteria transplantation group.

As exhibited in **Figure 7A**, the Faith_pd index in MADG + FMT group obviously increased compared with MADG-II group, while the Goods_coverage index showed no significant differences among three groups. The PCoA analysis revealed a significant separation among three groups, as the dots were observed to be widely dispersed (**Figure 7B**). Upon utilizing the Venn diagram method to analyze these three groups, a total of 510 common OTUs were identified. Furthermore, the MADG-II, MADG + LAP and MADG + FMT groups displayed unique OTU numbers of 6,719, 14,427 and 16,487, respectively (**Figure 7C**). At phylum level, Firmicutes/Bacteroidetes ratio significantly decreased in MADG + LAP and MADG + FMT groups compared to MADG-II group (**Figure 7E**). At genus level, the top 20 microorganism populations were analyzed (**Figure 7D**). And the main dominant bacteria were *Lactobacillus*, accounting for 6.05%, 8.39% and 6.58% in the MADG-II, MADG + LAP and MADG + FMT groups, respectively (**Figure 7F**). The abundance of *Lactobacillus*, *Prevotella and Allobaculum* were increased in MADG + LAP and MADG + FMT groups, whereas the abundance of *Oscillospira*, *Coprococcus*, *Bacteroides*, *Ruminococcaceae_Ruminococcus* and *Turicibacter* were decreased in MADG + LAP and MADG + FMT groups compared to MADG-II group.

## Discussion

EOC is the most common histopathological type of OC. In 2022, about 12,080 women died from OC in US, placing a heavy burden on both society and healthcare system (Siegel et al., 2022). While EOC patients usually respond well to primary cisplatin‐based chemotherapy, the majority eventually experience relapse and develop resistance to chemotherapy, leading to treatment failure and high mortality rates. Thus, discovery of innovative sensitizer drugs that can augment the efficacy of chemotherapy is crucial. Natural products have been long recognized as a wealthy source for new anticancer agent discovery. GTW, which is extracted from the root of *Tripterygium wilfordii* Hook F, has been used for inflammatory and autoimmune diseases for centuries. And recently, the antitumor effect of GTW has attracted extensive attention from scientific community.

Our previous studies have shown that GTW can inhibit proliferation, migration, and invasion abilities and intensify the sensitivity to A2780/DPP and SKOV3/DDP cells toward DDP *in vitro* and *in vivo* (Feng et al., 2021; Yu et al., 2022). Despite these findings, the precise mechanism underlying GTW’s anticancer properties remained to be fully elaborated. To further verify the anticancer activity and explore the underlying mechanism of GTW for EOC, we constructed a mouse xenograft ID8 cell line model. Our data suggested that GTW alone is unable to suppress ovarian tumor growth. However, when GTW is used in conjunction with DDP, it demonstrates a cisplatin-sensitizing effect similar to our prior study. Furthermore, our research results has indicated that the combined treatment of GTW and DDP can restore CA125 and HE4, two serum biomarkers associated with OC, to normal levels. Similarly, H&E staining of tumor tissues revealed that the combination treatment group resulted in the most significant tumor necrosis compared with the DDP treatment group. PCNA, a prognosis marker in diverse cancers, and some studies demonstrated that the elevated expression of PCNA is linked with the malignancy of EOC (Yang et al., 2019a). Our research utilized immunohistochemistry to reveal that the combined treatment of GTW and DDP can significantly reduce PCNA expression in tumor tissues compared to DDP treatment alone, indicating that the combination of GTW and DDP may inhibit tumor proliferation.

Moreover, IL-6 plays a central role in promoting inflammation and cancer promotion in OC, making it an attractive therapeutic target. A recent study suggested that OC patients with high peritoneal fluid IL-6 levels had poor overall survival rate and worse prognosis than patients with low IL-6 levels (Rodrigues et al., 2020). In addition, NF-κB/IL-6/STAT3 axis plays a key role in cancer chemotherapeutic resistance, where NF-κB can activate IL-6, which then can subsequently activate STAT3 through its receptor (Grivennikov and Karin, 2010). Both NF-κB and STAT3 are crucial in inflammation and tumor growth. Previous studies have demonstrated that the suppression of NF-κB and STAT3 signaling could inhibit the OC cells proliferation, invasion, as well as sensitized OC cells to cisplatin (Tian et al., 2019). We observed a significant suppression of protein levels for IL-6, phospho-NF-κB and p-STAT3 with the combined treatment of GTW and DDP, suggesting that GTW may display a chemotherapy sensitization effect on cisplatin by inhibiting the NF-κB/IL-6/STAT3 signaling pathways.

Cisplatin is known to have gastrointestinal toxicity, which may cause severe nausea, vomiting and gastrointestinal mucositis. Recent research has shown that the main active extract of GTW can alleviate the symptoms associated with colitis, including diarrhea, bloody stools, body weight loss, colonic atrophy and histopathological changes (Tang et al., 2020). Similar to Tang’s study, we detected histopathological changes and altered expression level of tight junction proteins in each group, revealing that GTW can reverse the increment in intestinal permeability and colon epithelial damage caused by cisplatin. These findings suggested that GTW not only has a chemotherapy sensitization effect, but also can reduce gastrointestinal side effects elicited by cisplatin.

Recently, accumulating studies have highlighted the critical role of a stable intestinal microbiota in chemotherapy effectiveness. For instance, a retrospective cohort study of EOC patients uncovered that using antibiotics during platinum-based chemotherapy may be correlated with poor prognosis (Chambers et al., 2020). Moreover, *in vivo* studies have also demonstrated that antibiotic-induced disruption of intestinal homeostasis can promote the growth of EOC and suppress cisplatin sensitivity (Chambers et al., 2022). Our study found that tumor growth was promoted in each single drug group when combined with antibiotic supplement, suggesting that eliminating gut microbiota can accelerate tumor progression. Additionally, our results shown that the cisplatin chemotherapy sensitizing effect and intestinal protection activity of GTW was diminished when mice were administered GTW, DDP and antibiotics concurrently.

To further understand the effect of GTW on gut microbiota, 16S rRNA high throughput sequencing was performed to analyze the changes in bacterial community structure. Through the Faith_pd index and Goods_coverage index, we found that treatment with GTW and DDP increased the α-diversity of gut microbiota compared to MA group. Previous studies have reported that the ratio of Proteobacteria/Firmicutes is elevated in OC patients relative to non-cancer patients (Zhou et al., 2019). Analyzing microbiota changes at the phylum and genus levels revealed that the ratio of Firmicutes/Bacteroidetes was significantly increased in MA group compared to MC group whereas was restored to MC group levels in MADG-I group. Notably, MADG-I group exhibited a significant enrichment of *Lactobacillus*, *Bacteroides* and *Allobaculum*, with *Lactobacillus* being the most abundant genus.

Previous research has identified *L. acidophilus* as having the potentials to exhibit anti-inflammatory properties, alleviate oxidative stress, and regulate immune responses(Gao et al., 2022). These benefits are particularly relevant in cases where immune function may be compromised due to cisplatin treatment. Additionally, *L. acidophilus* has shown promising in restoring imbalances in gut microbiota and maintaining the integrity of the intestinal barrier(Kang et al., 2022). Given its inclusion in list of probiotic strains approved by the China Ministry of Health, *L. acidophilus* becomes an attractive choice for potential utilization in future clinical trials. As a safe microorganism in food industry, *L. acidophilus* has also been reported can prevent severe diarrhea in cervical cancer patients receiving chemotherapy (Linn et al., 2019). Therefore, we assumed that *L. acidophilus* may act as a key microbiota and be responsible for the anticancer benefits of GTW. To test this, we performed FMT and *L. acidophilus* administration in MADG-II group mice and found that either FMT or *L. acidophilus* treatment exhibited a good therapeutic effect in MADG-II group; MADG + FMT group had a more potent antitumor effect and protective effects on intestinal barrier compared with MADG + LAP group. This result is similar to the findings of previous research conducted by Jotham Suez et al., who found that FMT can lead to a quicker and more comprehensive recovery of gut microbiota compared to multi-strain probiotics supplementation(Suez et al., 2018). There may be other probiotics play an active role in FMT that possess antitumor effects and *L. acidophilus* may be the keystone bacteria for GTW to halt EOC progression.

In further investigation, we performed 16S rRNA high-throughput sequencing on these three groups. *Prevotella* are known to be related with colitis in mice, which can exacerbate intestinal inflammation (El Hage et al., 2019). Unlike previous studies, the changes concerning the abundance of *Prevotella* in MADG + LAP and MADG + FMT groups were opposite to the changes in colon inflammation. Moreover, some studies have reported that the Wumei Decoction, a traditional Chinese herbal medicine, can increase the abundance of *Allobaculum* to ameliorate chronic colitis in mice (Wu et al., 2022). Our study also found that *L. acidophilus* supplementation and FMT can substantially recover the abundance of *Allobaculum*. Thus, the mechanism of GTW in treating EOC may be related to reducing colon inflammation, maintaining colon microbial homeostasis and repairing the intestinal barrier.

In addition, the main limitation of this study is lack of the evaluation of how GTW administration affects liver and kidney function, as well as its potentials in immunosuppression. While herbal Traditional Chinese Medicine is generally assumed to be safe, it is important to consider potential side effects, particularly during long-term treatment. Another limitation is the small number of mice utilized in our experiment. To adequately assess the viability of GTW for human clinical trials, it is essential to conduct future studies with a larger cohort of animals. Although pseudo germ-free mice offer a cost-effective alternative to germ-free mice, our study revealed that the use of antibiotics had detrimental effects on tumors, and it even resulted in the death of some mice. Using germ-free mice in future experiments may effectively overcome the issue. Additionally, our study primarily focuses on observing the effects of *L. acidophilus* in enhancing chemosensitivity and protecting the intestinal barrier. However, it should be noted that our exploration of the underlying mechanism is still in its preliminary stages. In future research endeavors, we will prioritize investigating the mechanisms underlying the observed effects.

## Conclusion

In conclusion, our findings suggested that GTW combined with DDP can restore intestinal microbial imbalance, downregulate the activated NF-κB/IL-6/STAT3 signaling pathway and repair damaged intestinal barriers. Collectively, our results indicated that GTW holds promise as an adjuvant chemotherapy agent, capable of enhancing cisplatin’s anticancer effects while reducing its associated gastrointestinal side effects.

## Data availability statement

The datasets presented in this study can be found in online repositories. The names of the repository/repositories and accession number can be found below: PRJNA778571 (SRA).

## Ethics statement

All animal experiments were ratified by the Laboratory Animal Ethics Committee of Nanchang Royo Biotechnology Co. Ltd (Ethics Number: RYE2021022501). The study was conducted in accordance with the local legislation and institutional requirements.

## Author contributions

XZ performed experiments, analyzed the data, and wrote the paper. QZ conceived and designed experiments. GH, ZQ, SZ, YZ, and YX discussed the results. YW reviewed and polished the manuscript. TC and BT led the overall direction of the project. All authors contributed to the article and approved the submitted version.
