# Correction: *Tripterygium* glycosides sensitizes cisplatin chemotherapeutic potency by modulating gut microbiota in epithelial ovarian cancer

**DOI:** 10.3389/fcimb.2026.1797134

**Published:** 2026-02-25

**Authors:** Xinlu Zhan, Qi Zuo, Genhua Huang, Zhanghua Qi, Yufan Wang, Sihong Zhu, Yanying Zhong, Yifei Xiong, Tingtao Chen, Buzhen Tan

**Affiliations:** 1 Department of Obstetrics & Gynecology, The Second Affiliated Hospital of Nanchang University, Nanchang, China; 2 Department of Obstetrics & Gynecology, Ji’an Central People’s Hospital, Ji’an, China; 3 Institute of Translational Medicine, Nanchang University, Nanchang, China

**Keywords:** GTW, gut microbiota, chemosensitization, *Lactobacillus acidophilus*, intestinal barrier

There was a mistake in **Figure 1** and **Figure 3** as published. In **Figure 1G**, **3G**, some images contain duplicated areas. Specifically, these occur between: **Figure 1G** (representative immunohistochemical expression of P-STAT3 in tumor tissues of the MDG group) and **Figure 2G** (representative immunohistochemical expression of P-STAT3 in tumor tissues of the MDG group); **Figure 2G** (representative immunohistochemical expression of PCNA in tumor tissues of the MADG-I group) and **Figure 3G** (representative immunohistochemical expression of PCNA in tumor tissues of the MADG-II group). The corrected **Figure 1** and **Figure 3** appear below.

**Figure 1:**
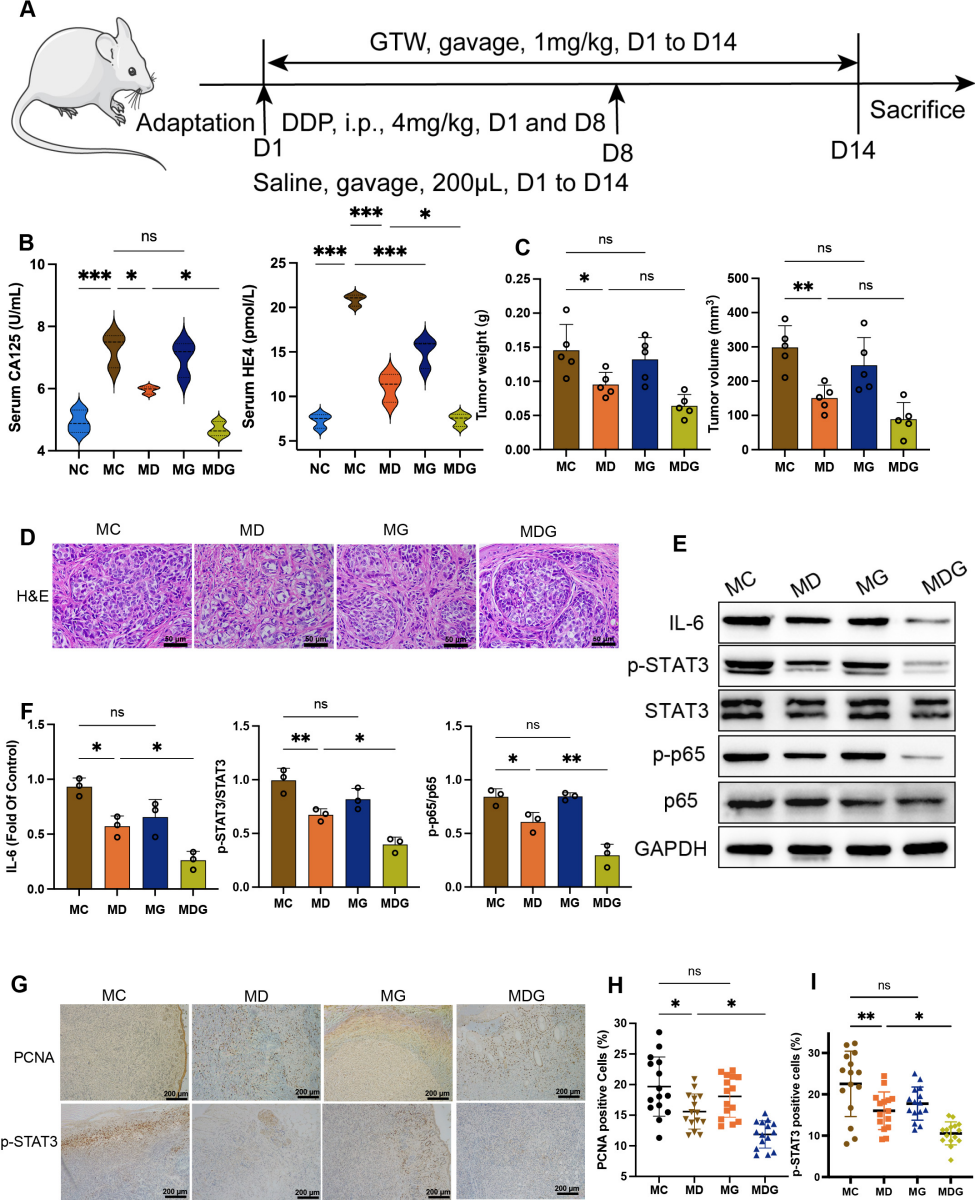
**(A)** Schematic of schedule of whole experiment. **(B)** CA125 and HE4 level in serum (n = 3). **(C)** Weights and volumes of tumours in different treatment groups. **(D)** Representative H&E staining image in tumour tissues (400x). Scale bsars, 50 µm. **(E)** Effect of different drugs on the protein expression in tumour tissues (n = 3). **(F)** Relative expression of IL-6, p-STAT3/STAT3 and p-p65/p65 in tumour tissues by ImageJ software (n = 3). **(G)** Representative immunohistochemical expression of PCNA and p-STAT3 in tumour tissues (100x). Scale bars, 200 µm. **(H, I)** Statistic analysis of PCNA and p-STAT3 positive cell expression in different drug groups. Five fields of three tumour sections from each group were randomly selected and examined. *p < 0.05, **p < 0.01, ***p < 0.001. NC stands for normal control group, MC stands for model control group, MD stands for model + DDP group, MG stands for model + GTW group, MDG stands for model + DDP + GTW group. ns, no significance.

**Figure 3:**
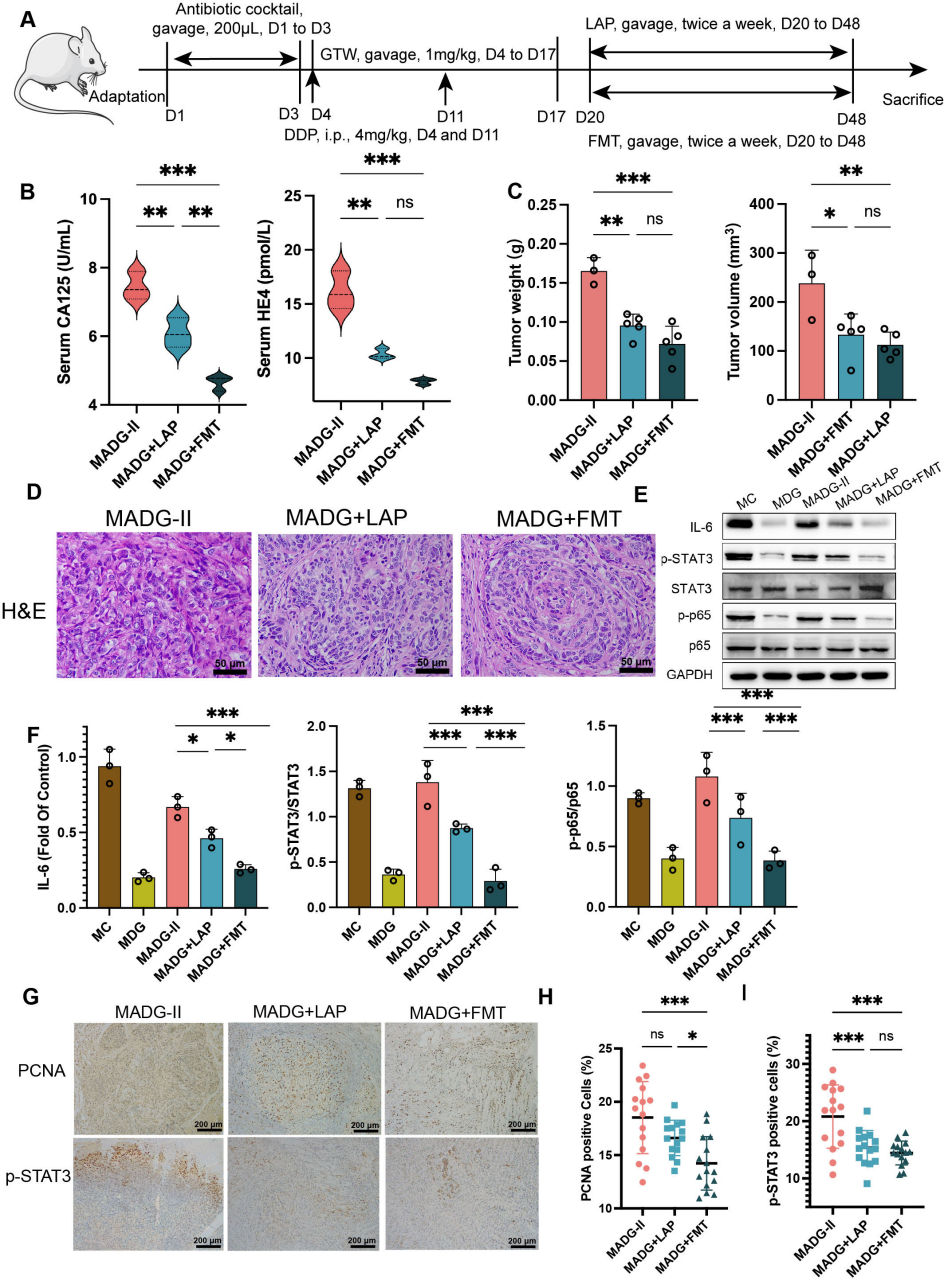
**(A)** Schematic of schedule of whole experiment. **(B)** CA125 and HE4 level in serum (n = 3). **(C)** Weights and volumes of tumours in different treatment groups. **(D)** Representative H&E staining image in tumour tissues (400x). Scale bars, 50 µm. **(E)** Effect of different drugs on the protein expression in tumour tissues (n = 3). **(F)** Relative expression of IL-6, p-STAT3/STAT3 and p-p65/p65 in tumour tissues by ImageJ software (n = 3). **(G)** Representative immunohistochemical expression of PCNA and p-STAT3 in tumour tissues (100x). Scale bars, 200 µm. **(H, I)** Statistic analysis of PCNA and p-STAT3 positive cell expression in different drug groups. Five fields of three tumour sections from each group were randomly selected and examined. *p < 0.05, **p < 0.01, ***p < 0.001. MADG-II stands for model + antibiotic +DDP + GTW group, MADG + LAP stands for model + antibiotic + GTW + DDP + L. acidophilus group, MADG + FMT stands for model + antibiotic + GTW + DDP + fecal bacteria transplantation group. ns, no significance.

The original version of this article has been updated.

